# The Effects of Shift Work on Cardio-Metabolic Diseases and Eating Patterns

**DOI:** 10.3390/nu13114178

**Published:** 2021-11-22

**Authors:** Alexandra Hemmer, Julie Mareschal, Charna Dibner, Jacques A. Pralong, Victor Dorribo, Stephen Perrig, Laurence Genton, Claude Pichard, Tinh-Hai Collet

**Affiliations:** 1 Nutrition Unit, Service of Endocrinology, Diabetes, Nutrition and Therapeutic Education, Department of Medicine, Geneva University Hospitals (HUG), 1211 Geneva, Switzerland; alexandra.hemmer@hcuge.ch (A.H.); julie.mareschal@hcuge.ch (J.M.); laurence.genton@hcuge.ch (L.G.); claude.pichard@hcuge.ch (C.P.); 2 Service of Endocrinology, Diabetes, Nutrition and Therapeutic Education, Department of Medicine, Geneva University Hospitals (HUG), 1211 Geneva, Switzerland; charna.dibner@unige.ch; 3 Department of Cell Physiology and Metabolism, Faculty Diabetes Center, Faculty of Medicine, University of Geneva, 1211 Geneva, Switzerland; 4 Service of Pneumology, Department of Medicine, Geneva University Hospitals (HUG), 1211 Geneva, Switzerland; jacques-andre.pralong@hcuge.ch (J.A.P.); stephen.perrig@hcuge.ch (S.P.); 5 Department of Occupational and Environmental Health, Center for Primary Care and Public Health (Unisanté), University of Lausanne, 1066 Épalinges, Switzerland; victor.dorribo@unisante.ch; 6 Faculty of Medicine, University of Geneva, 1211 Geneva, Switzerland

**Keywords:** shift work, circadian misalignment, cardiovascular disease, metabolic disease, eating patterns

## Abstract

Energy metabolism is tightly linked with circadian rhythms, exposure to ambient light, sleep/wake, fasting/eating, and rest/activity cycles. External factors, such as shift work, lead to a disruption of these rhythms, often called circadian misalignment. Circadian misalignment has an impact on some physiological markers. However, these proxy measurements do not immediately translate into major clinical health outcomes, as shown by later detrimental health effects of shift work and cardio-metabolic disorders. This review focuses on the effects of shift work on circadian rhythms and its implications in cardio-metabolic disorders and eating patterns. Shift work appears to be a risk factor of overweight, obesity, type 2 diabetes, elevated blood pressure, and the metabolic syndrome. However, past studies showed discordant findings regarding the changes of lipid profile and eating patterns. Most studies were either small and short lab studies, or bigger and longer cohort studies, which could not measure health outcomes in a detailed manner. These two designs explain the heterogeneity of shift schedules, occupations, sample size, and methods across studies. Given the burden of non-communicable diseases and the growing concerns about shift workers’ health, novel approaches to study shift work in real contexts are needed and would allow a better understanding of the interlocked risk factors and potential mechanisms involved in the onset of metabolic disorders.

## 1. Introduction

Most of the labor force in Europe work between 08:00 and 19:00 from Monday to Friday, but an increasing number of workers do not follow these office working hours [1]. In the last pre-COVID report of the European Working Conditions Survey, only 43% of workers have regular working schedules, whereas 21% work mainly in rotating or permanent shifts. More specifically, night shift concerns 19% of workers. Moreover, the proportion of individuals with atypical working hours has increased over the last decade [1,2].

Shift work is defined in Europe as working outside office working hours (i.e., 9:00 until 17:00 five days a week) on different shifts patterns, whereas night work comprises working at least two hours between 22:00 and 05:00 [1]. The definitions of shift work vary a lot between surveys and countries, because they are usually based on a local legal definition [1], rather than a scientific background. This explains the heterogeneity between study results [1,2]. The structure of shift work and night work schedules also vary, but a common delineation differentiates permanent shift work (always on night shifts) from rotating shift work (a rotation between day shifts, evening shifts, and night shifts), either in a regular or an irregular schedule structure. Interestingly, some countries have integrated a notion of regularity/frequency of night shifts over the entire year [3].

Shift work has long been associated with negative health outcomes, such as increased risk of cardiovascular disease (CVD) and metabolic diseases, fatigue and sleep disorders, mood disorders, gastrointestinal disorders, and increased risk of miscarriage and premature birth [1,4,5,6,7,8]. Besides, night shifts are considered “probably carcinogenic to humans”, based on limited evidence in humans but stronger evidence in animals by the International Agency for Research on Cancer [9]. Shift work also has an impact on socio-professional outcomes, such as family organization, social activities, increase of errors and accidents at the workplace, or decreased work performance [8]. This review mainly focuses on circadian misalignment induced by shift work and its metabolic effects, and the associated changes in eating patterns. Given the lack of consensus and the heterogeneity of definitions of shift work and studies on the topic, we summarized in Table 1 the characteristics (study population and design, types and definition of shift work) and main outcomes (cardio-metabolic and eating patterns) of the studies reviewed in Section 3 and Section 4. This could provide leads for a further standard definition and potential consensus in the field.

## 2. Circadian Disruption and Its Impact on Human Physiology

The circadian rhythms have an approximate period of 24 h and allow the biological and physiological systems to anticipate the light/dark cycle of the 24 h environment. In normal circumstances, the diurnal phase is aligned with ambient light and prepares energy metabolism to diurnal activity and eating, while the nocturnal phase with low intensity of light is in principle dedicated to sleep and fasting. External factors, such as shift work, lead to a disruption of the inner clocks, often called *circadian disruption* or *circadian misalignment*, with near overlap of the terms [6].

Even though circadian misalignment has an impact on some physiological markers, such as hormone levels, blood pressure, and immune markers [11,13], most individuals do not notice these changes unless measured specifically. Thus, these proxy measurements do not immediately translate into clinical symptoms of circadian misalignment [53]. However, by inducing circadian misalignment, shift work can have a detrimental effect on health with long-term consequences, such as obesity, metabolic disorders, CVD, and other conditions (Section 1).

By disturbing the relationship between environmental cues and biological rhythms, shift work is an interesting model to study circadian misalignment [54]. Most studies have either been small and short experimental human studies, or large-scale epidemiological studies with limited assessment of the underlying physiological mechanisms and limited information on the exposure to shift work [6,53]. Controlled human lab studies mimicking night shift work with forced circadian desynchronization protocols studied *short-term* circadian misalignment, whereas *long-term* effects have mostly been assessed in cohort and field studies in real-life settings with a wide heterogeneity of individual cycles (sleep/wake, fasting/eating, rest/activity cycles), population characteristics (e.g., schedules and occupation), and study methods 

A proper definition of shift work (including frequency, duration, and schedule structure) and cut-offs to distinguish short-term and long-term misalignment as well as the chronicity of shift work remain elusive [6]. Studies on chronic shift work often consider at least 12 months of exposure [10,13,14,15,18,33]. However, the frequency/regularity of shifts and the annual number of night shifts is rarely reported in shift work studies. Although a common terminology has not been agreed upon in the literature, we will consider the effects of *short-term circadian misalignment* when exposure is <1 month, the *acute effects of shift work* <12 months, and the *chronic effects of shift work* when exposure lasts >12 months. In this review, we could not integrate the notion of frequency/regularity in our definition of shift work.

Shift workers are more likely to develop cardio-metabolic disorders, especially after recurrent or prolonged exposure to shift work, but the possible underlying mechanisms are multiple and interlocked [6]. Shift work could contribute to metabolic disorders by disrupting the alignment of biological and environmental rhythms, such as inducing sleep deprivation [55], decreasing the resting metabolic rate [56], physical activity, daily energy expenditure during night shifts and in response to dinner [10], and changes in dietary habits and behavior [55].

## 3. Cardio-Metabolic Effects

Shift work has a deleterious effect on multiple cardiovascular risk factors leading to the development of chronic diseases, such as CVD and type 2 diabetes (T2D), often referred to as cardio-metabolic diseases. We will first detail the effects of shift work on the metabolic syndrome (MetS) as a whole entity [57] here, then separately for each component of the MetS (abdominal obesity, impaired glucose tolerance, dyslipidemia, and hypertension) in the following subsections.

In a meta-analysis of 13 studies, MetS was associated with ‘ever being exposed to shift work’ (relative risk [RR] 1.57, 95% confidence interval [95%CI] 1.24–1.98) [38]. They reported a potential dose–response relationship, i.e., an increased risk with longer exposure to shift work (RR 1.77, 95%CI 1.32–2.36 after ≥10 years of night shifts). Recently, another meta-analysis of 38 studies confirmed an increased risk of MetS among rotating shift workers (odds ratio [OR] 1.29, 95%CI 1.11–1.46) [39]. Similar results across 36 studies were reported by Wang et al. (OR 1.42, 95%CI 1.18–1.71) [40]. However, the studies included in these meta-analyses relied on heterogeneous study designs, outcome definitions, various exposure to shift work, and unmeasured confounding factors.

A prospective cohort over 6 years found a greater risk of developing MetS (OR 1.77, 95%CI 1.34–2.32, adjusted OR 1.46, 95%CI 1.04–2.07) and each of its component independently in rotating shift workers compared to day workers [28]. Besides, they reported a positive association between the duration of shift work exposure and MetS risk.

### 3.1. Overweight and Obesity

Shift work appeared to be a risk factor for overweight and obesity in a meta-analysis of 28 studies (OR 1.23, 95%CI 1.17–1.29) [41]. Another meta-analysis of 26 studies confirmed this association between shift work and overweight (RR 1.25, 95%CI 1.08–1.44), as well as obesity (RR 1.17, 95%CI 1.12–1.22) [42]. More specifically, this association was more pronounced for abdominal obesity (OR 1.35, 95%CI 1.13–1.61) [41]. However, another meta-analysis of six cross-sectional studies of shift-working nurses did not find an increased prevalence of obesity [43].

While most studies agree on the association between shift work and overweight/obesity, the results are less clear regarding the type of schedules, probably due to the heterogeneity of definitions and schedules patterns. In a Chinese cohort of industrial workers, permanent night workers (i.e., night shifts from 24:00 to 6:00 once a month during >1 year) and irregular night shift workers (i.e., night shifts without a fixed schedule) were at higher risk of overweight (adjusted OR 3.94, 95%CI 1.40–11.03, and OR 1.56, 95%CI 1.13–2.14, respectively) and obesity (adjusted OR 3.34, 95%CI 1.19–9.37, and OR 1.26, 95%CI 0.94–1.69, respectively) compared to rotating shift workers [33].

In their meta-analysis of 26 studies, Liu et al. reported that night shift workers had a higher risk of overweight (RR 1.38, 95%CI 1.06–1.80) than rotating shift workers, similarly for obesity (RR 1.18, 95%CI 1.08–1.29) [42]. These results were consistent with a cohort of Norwegian nurses where night shift workers had an increased body mass index (BMI) compared to day workers [34]. In two large cohorts of American nurses, those working on rotating night shifts (i.e., at least 3 nights per month in addition to days and evenings in the month) were more likely to gain weight over the 18–20 years of follow-up [30].

Furthermore, the duration of exposure, frequency, and intensity of night shift work seems to be a determinant in the development of overweight and obesity, as suggested by Sun et al. [41]. A positive association between the duration of shift work and the prevalence of overweight or obesity has been proposed in several cohort studies [23,30,33]. Ramin et al. reported an association between the exposure to night shifts and the risk of obesity, suggesting that the number of night shifts (from 23:00 to 07:00) per month might be a risk factor for obesity [29]. On the contrary, in the cohort of Norwegian nurses, no association was found between the number of night shifts per year and BMI evolution [34]. The authors suggested a potential survivor effect, i.e., only those tolerating night shift on the long run would keep on this work schedule, or a selection bias in the cohort.

### 3.2. Glucose Metabolism

The impact of circadian misalignment on glucose metabolism has mostly been studied in carefully controlled lab studies. In an 8-day crossover study of healthy non-shift workers, Morris et al. showed that circadian misalignment increased postprandial glucose levels by 6% with no difference in fasting glucose levels [12]. The same team found similar results in a 3-day crossover lab study of chronic shift workers [14]. Both studies suggested decreased insulin sensitivity during circadian misalignment. Sharma et al. enrolled rotational shift workers performing a minimum of 3 night shifts per month for at least 1 year, a population similar to the one studied by Morris et al., and found a higher postprandial glucose excursion during the night shift [15]. Bescos et al. reported a decrease in insulin sensitivity after four successive simulated night shifts in healthy adults [16].

While some studies reported no difference in fasting glucose levels [12,14,15], others found an increase of fasting glucose levels [16,56]. These discrepant results may be due to differences in the study design (simulated shift work vs. sleep restriction protocol) or duration of the tested intervention (from days to weeks). Overall, these results suggest that circadian misalignment induced by simulated shift work may contribute to the reduced glucose tolerance [58,59].

Several studies reported an increased risk of T2D in shift workers with a dose–response relationship with the duration of exposure to shift work [30,44,45]. In two large cohorts of American nurses, this increased risk of T2D remained significant after adjustment for BMI [30]. A meta-analysis of 12 cohort studies not only confirmed this association (RR 1.14, 95%CI 1.10–1.19), but also reported a correlation between the exposure to shift work and the risk of T2D in women (per 5-year increase in exposure: RR 1.07, 95%CI 1.04–1.09) [44]. The latter analysis included only female cohorts. In another meta-analysis of 21 cohort and cross-sectional studies, Gao et al. found that shift workers were more likely to develop T2D than day workers (RR 1.10, 95%CI 1.05–1.14), with a higher risk for night and rotating shifts. They also found an association between the number of years of exposure to shift work and T2D (per 5-year increase in exposure: RR 1.05, 95%CI 1.03–1.07) [45].

In a cohort of Danish nurses, Hansen et al. found that evening shifts and night shifts increased the risk of T2D (hazard ratio [HR] 1.29, 95%CI 1.04–1.59 and HR 1.58, 95%CI 1.25–1.99, respectively) compared to day shifts after adjustment for age, BMI, CVD, and lifestyle factors [32]. However, they found no significant risk for rotating shift work.

Finally, Shan et al. found an association between rotating shift and T2D among American nurses [31]. More specifically, the risk of T2D increased with the duration of rotational shift work, i.e., the HR increased from 1.04 (95%CI 1.00–1.09) with 1-5 years of shift work to 1.16 (95%CI 1.09–1.24) after ≥10 years with multiple adjustments including BMI.

### 3.3. Cardiovascular Risk, Blood Pressure, and Lipid Profile

Shift work increases the risk of CVD and CVD-related mortality [7,60]. In a meta-analysis of 21 studies, shift workers were more likely to develop CVD events (RR 1.17, 95%CI 1.09–1.25), thus leading to an increased CVD mortality (RR 1.22, 95%CI 1.09–1.37) [46]. More specifically, the risk of coronary heart disease (CHD) (RR 1.26, 95%CI 1.10–1.43) and CHD-related mortality (RR 1.18, 95%CI 1.03–1.32) was higher among shift workers than non-shift workers. The authors reported a dose–response relationship between the duration of shift work and CVD risk, rising significantly after the first 5 years of exposure to shift work (for every additional 5 years: RR 1.07, 95%CI 1.05–1.10).

The effect of circadian misalignment on blood pressure (induced by shift work or simulated) has been studied both in cohorts and in human controlled lab studies with crossover designs. A meta-analysis of 27 studies, including 9 cohorts, reported that shift workers were more likely to have hypertension (OR 1.31, 95%CI 1.07–1.60) [47]. In their 8-day crossover study simulating short-term circadian misalignment in healthy non-shift workers, Morris et al. found increased systolic and diastolic blood pressure after night shifts, and reduced heart rate variability, reflecting a decrease in vagal stimulation [11]. In a related 3-day crossover lab study in chronic shift workers, Morris et al. confirmed the increased 24 h ambulatory blood pressure, in addition to higher high-sensitivity C-reactive protein in circadian misalignment [13].

To explore the relationship of shift work with dyslipidemia, Dutheil et al. reviewed 66 articles and found increased levels of triglycerides (overall standard mean difference [SMD] 0.09, 95%CI 0.05 to 0.13) and decreased levels of high-density lipoprotein cholesterol (SMD −0.08, 95%CI −0.12 to −0.03) among shift workers but no significant difference for low-density lipoprotein (LDL) cholesterol [48]. The changes seemed greater among permanent night workers for total cholesterol and triglycerides. In a cohort of Dutch healthcare workers, Loef et al. found lower levels of total and LDL cholesterol in night shift workers compared to non-shift workers (difference in total cholesterol −0.38 mmol/L, 95%CI −0.73 to −0.04; LDL cholesterol −0.34 mmol/L, 95%CI −0.60 to −0.08) [36]. However, the age, occupation, and education were different between shift workers and non-shift workers, which could partly explain these differences. In another Dutch cohort, Hulsegge et al. did not find any difference in blood pressure and lipids between shift workers and non-shift workers [37]. These discordant effects of shift work on the lipid profile and the confounding factors need to be addressed in future studies.

## 4. Sleeping and Eating Patterns in Shift Work

In addition to the changes of cardiovascular risk factors, circadian misalignment can disrupt biological and environmental rhythms, such as the sleep/wake and the fasting/eating cycles.

### 4.1. The Impact of Sleep/Wake Cycles on Energy Balance and Eating Behavior

Several studies have investigated the impact of sleep on eating patterns. Most studies and meta-analyses of healthy adults reported that shorter sleep duration was associated with an increase of the total energy intake [61,62,63]. However, their findings were limited and often divergent regarding the intake of specific macronutrients.

Most studies found an increased intake of fat and decreased intake of protein among short sleepers (≤6 h) [61,64]. Both papers reported an association between short sleep duration, nutritional quality, and irregular eating behavior. In addition, Dashti et al. suggested that short sleepers consumed irregular highly palatable (energy-dense) meals and snacks [61].

In a lab study restricting sleep to 5 h for 5 nights, sleep deprivation increased total energy expenditure by 5% but with a greater increase of total food intake, thus leading to a positive energy balance and a modest weight gain [65]. In their meta-analysis of 11 studies, Al Khatib et al. described the association of sleep deprivation and positive energy balance [62]. They found no effect on energy expenditure, but the impact on weight was not specified.

Interestingly, in a study restricting calorie intake to 10% of energy requirements for 48 h, the duration of deep sleep increased and reverted to baseline after energy intake was restored [66]. This study highlighted a potential bidirectional relationship between sleep duration and energy intake, which needs further study.

### 4.2. The Impact of Shift Work on Energy and Macronutrient Intake

While there are several studies on the links between sleep restriction and positive energy balance (Section 4.1), only limited evidence exists on the specific effects of shift work on eating patterns. By extension, changes in the metabolic parameters (Section 3) and eating patterns of shift workers might be mediated by sleep restriction induced by shift work [67]. In addition, potential biases need to be considered while reviewing these studies, such as data collection and dietary assessment, adjustments for potential confounding factors, the heterogeneous designs and studied populations, as well as the sample size [68,69].

Most studies reported no difference in *energy intake* between shift workers and day workers [17,20,21,49,68]. The comparison of shift schedules (early morning, day, afternoon/evening, or night shifts) showed no difference in energy intake either [50]. Interestingly, Flanagan et al. described no difference in the total energy intake among shift workers during their night shift or non-night shift, but the energy intake was redistributed during the night shift [26]. On the contrary, a few studies found an increased energy intake among shift workers [19,24,29]. For instance, Pepłońska et al. found an adjusted mean energy intake of 2005 kcal (95%CI 1928–2084) among rotating shift workers vs. 1850 kcal (95%CI 1782–1921) among day shift workers (*p* = 0.007) [24]. However, these studies do not rely on the same methodologies for dietary assessment (e.g., smartphone-based food diary and a wearable device detecting the bite counts [19], food frequency questionnaire [24,29]) and no hard conclusions can be drawn until further studies.

Furthermore, only a few studies investigated *macronutrient intake* among shift workers and the results were most often discrepant [49]. The systematic review by Cayanan et al. did not find any difference in macronutrient intake during night shift workers compared to non-shift workers [50]. In a comparison of different shifts of American healthcare workers, Chen et al. showed an association between shift work and calorie intake with higher fat and carbohydrate intake [19]. Similarly, among Polish healthcare professionals, daily fat and carbohydrate intake were significantly increased on rotating night shifts compared to day shifts (adjusted mean 78 g/day, 95%CI 74–82, vs. 70 g/day, 95%CI 67–74 for fat; 266 g/day, 95%CI 254–278 vs. 244 g/day, 95%CI 233–254 for carbohydrates) [24].

In a cross-sectional study, Chen et al. compared night and day healthcare shift workers after a series of two to three consecutive work shifts [20]. They found a lower proportion of protein consumption during the meal at the lab in the night shift group but no difference in energy and macronutrients intake between groups. In another lab study of non-shift workers, Cain et al. reported no difference in total calorie, protein, or carbohydrate consumption after a simulated night shift but demonstrated an increased preference for high-fat content foods [17].

### 4.3. The Impact of Shift Work on Eating Behavior and Nutritional Quality

While most studies agree that shift work leads to changes in sleeping patterns [19,20,21,29] and that sleep affects eating patterns (Section 4.1), the influence of shift work itself on *eating behavior* remains elusive [67]. Shift work may impact the food type and content as well as the eating behavior. However, only few studies have assessed the specific effects of shift work.

In their review, Souza et al. found a greater consumption of *unhealthy foods*, such as saturated fat and soft drinks, among shift workers [51]. In an observational study of Swiss industry workers, Bucher della Torre et al. showed that even one night shift was associated with consumption of unhealthy food (as assessed with the adherence to the French Programme National Nutrition Santé Guideline Score [70]) across a large variety of shift schedules [25]. In a cross-sectional study of Polish rotating shift healthcare workers, Pepłońska et al. specifically identified a greater consumption of energy, fat, carbohydrates, and saccharose as well as a lower consumption of fruits and vegetables [24].

By using a device recording the timestamp of hand movements to the mouth, Chen et al. showed that night shift workers *snacked* more than day and rotating shift healthcare workers [19]. Similarly, in a cross-sectional study of Canadian nurses, Terada et al. reported an increased consumption of snacks in quantity, frequency, and quality [22].

Finally, in a Dutch cohort, Hulsegge et al. found opposed results, with no difference in meal and snack frequency, nor snack quality between shift workers and day workers [35]. However, snacking habits changed when the workers changed shifts. The contrasted findings may be influenced by several biases as described above (Section 4.2).

### 4.4. The Impact of Shift Work on the Timing of Eating

Besides the impact of shift work on nutrient intake, the type of consumed food, and eating behavior, shift work might also have an influence on the *timing of eating* [49,51]. However, this notion is loosely defined in the literature and is very variable between individuals.

Studies have shown that shift work is associated with *longer eating duration*, i.e., the time interval between the first and the last consumption in a 24 h period [18,21,22]. In an observational study of healthcare workers, those on night shifts self-reported a longer eating duration than day workers (mean 14.2 h ± SD 3.8 h vs. 12.0 h ± 1.5 h, respectively, *p* = 0.02) [21]. The fasting period was therefore shorter for shift workers (11.8 h ± 2.0 h vs. 13.3 h ± 1.9 h in non-shift workers, *p* = 0.02) [22]. In a randomized crossover trial, food consumption was nearly around the clock during night shifts compared to day shifts [18].

The *energy distribution during the 24-h period* can be disrupted by shift work [26,49,51,68,69] without necessarily an increase in energy intake. Multiple meta-analyses were performed and did not always find consistent results in energy distribution between night shifts and day shifts. This is probably due to the lack of adjustment for important covariates, unmeasured confounders, or differences in the shift structure and designs of the underlying studies [49,51,69].

Shift work also leads to *irregular meals* [22,68,69]. In their meta-analysis, Lowden et al. reported that night shift workers tended to maintain the three meals a day structure, but with less regular patterns especially at night [68]. Moreover, night and rotating shift workers tended to *skip meals* more frequently [51], and more specifically skip breakfast [23].

These findings on the temporal changes in eating behavior of shift workers explain the increased interest in chrononutrition [21,49,50] and chronotypes (i.e., the individual’s propensity to early sleep and activity, morningness vs. late sleep and activity, eveningness) [71]. The individual chronotype might also influence the tolerance to shift work and its impact on cardio-metabolic health [36,37,72,73], and eating behavior in the general population [74,75,76]. Gupta et al. reviewed the factors influencing the eating behavior of shift workers, highlighting that in addition to food content (the *what*), the timing of eating (the *when*), the environment and source of food (the *where*), and the reasons of eating during the shift (the *why*) are also part of the eating behavior of shift workers [52].

Yet another avenue could be the central regulation of eating behavior, whereby appetite-regulating hormones could control the hedonic pathways and thus play a role in the changes of eating patterns and in turn the metabolic effects of shift work [27,61,64,77]. However, these aspects are beyond the scope of this review.

## 5. Conclusions and Perspectives

Shift work is a risk factor of overweight, obesity, T2D, increased blood pressure, and MetS. As such, shift work could be considered alongside the classic cardiovascular risk factors. Some studies have explored the effects of different shift work schedules on the cardio-metabolic conditions but results vary. Taken alone, these changes due to shift work may appear small, but the long-term additive effects of exposure to multiple cardiovascular risk factors lead to CVD morbidity and mortality.

In addition, shift work impacts on eating behavior, food type and content, energy, and macronutrient intake. Given the limited number of studies and their heterogeneity in methodology (designs, studied population, data collection, dietary assessment, adjustments for potential confounding factors), the findings on the influence of shift work on eating patterns should be considered with caution and only hypothesis generating.

The impact of shift work is probably multifactorial and other factors might affect shift workers’ health, such as the intensity, timing, and patterns of physical activity; professional constraints and stress; anxiety; mental illness; sleep disorders; fitness and tolerance to shift work (potentially interacting with chronotype); and other yet unidentified factors. Given the burden of non-communicable diseases and the concerns about shift workers’ health, the assessment of these factors in real-life settings would allow a better understanding of the consequences of shift work on health and the intervening factors. In addition, a proper and accurate definition of shift work is required and has to be taken into account as a limitation of most studies in the field and the current review.

With the advent of mobile technologies and new research paradigms in clinical and translational research, further studies should rely on prospective designs and investigate the interlocked risk factors and mechanisms involved in the onset of metabolic disorders and the changes in eating patterns induced by shift work. The field needs standardized data collection and adjustment for covariates across studies. Thus, we suggest a minimal set of covariates, including age, sex, BMI, medical conditions, medication, work schedule (type, duration, and frequency/regularity of shifts), lifestyle (eating habits, physical activity, smoking, sleep, chronotype), and socio-familial factors, to be further extended according to the research question in future studies.

## Figures and Tables

**Table 1:** Population, design, shift work definition(s) and exposure, and main outcomes of the reviewed studies ^1^.

Ref.	Loc.	N.	Sex	Occupation	Design	Type of Shift Work	Exposure and Definition(s) of Shifts	Main Outcomes						
								MetS	BMI	Gluc.	CVD	BP	Lip.	Nut.
[10]	USA	14	Both	n.s.	Lab study	Simulated	Exposure: No shiftwork in the year prior to the study Simulated night shift: 16 h of wakefulness, 8 h of sleep opportunity		x					x
[11,12]	USA	14	Both	n.s.	Lab study, crossover	Simulated	Exposure: No shift work in the past 3 years and <6 months cumulative lifetime exposure to shift work Simulated night shift: Kept awake from 19:00 to 11:00, 8 h sleep opportunity from 11:00 to 19:00			x	x	x	x	
[13,14]	USA	9	Both	n.s.	Lab study, crossover	Simulated	Exposure: ≥12 months of consecutive shift work with ≥5 night shifts per month prior to the study Simulated night shift: Kept awake from 19:00 to 11:00, 8 h sleep opportunity from 11:00 to 19:00			x	x	x		
[15]	USA	12	Both	Healthcare	Lab study, crossover	Simulated	Exposure: Rotating shifts of 3 consecutive day and nights shifts (07:00–19:00 and 19:00–07:00, resp.), with a minimum of 3 night shifts per month for ≥1 year Simulated day and night shift following the same clock times			x				
[16]	AUS	17	Both	n.s.	Lab study	Simulated	Exposure: No shift work in the past 2 months Simulated day shift: Kept awake from 07:00 to 22:00, 9 h sleep opportunity from 22:00 to 07:00 Simulated night shift: Kept awake from 17:00 to 08:00, 9 h sleep opportunity from 08:00 to 17:00			x				
[17]	AUS	16	Both	n.s.	Lab study, crossover	Simulated	Exposure: No shift work prior to the study Simulated night shift: Kept awake from 20:00 to 08:00							x
[18]	AUS	22	Both	Private companies	Randomized crossover trial ^2^	Night shift work	Exposure: ≥12 consecutive months of nights (permanent, rotating, or split night shifts) prior to the study		x					x
[19]	USA	14	Both	Healthcare	Cross-sectional, prospective	Day, night, and rotating shift work	Exposure groups: Day, night, and rotating shift workers	x	x	x		x		x
[20,21]	USA	24	Fem.	Healthcare	Cross-sectional, prospective	Day and night shift work, without rotating shift work	Exposure groups: Day shift workers (from 07:00–08:00 to 15:00–20:00) vs. night shift workers (from 19:00–23:00 to 07:00); each shift lasting 8 h or 12 h		x			x		x
[22]	CAN	73	Fem.	Healthcare	Cross-sectional, secondary analysis ^3^	Evening and night shift work, including irregular or rotating schedules	Exposure groups: Shift workers (any work outside of daytime hours) vs. non-shift workers		x			x		x
[23]	KOR	9989	Fem.	Healthcare	Cross-sectional, prospective ^4^	n.s.	Exposure groups: Current shift workers vs. day workers		x					x
[24]	POL	522	Fem.	Healthcare	Cross-sectional, prospective ^5^	Rotating shift work	Exposure groups: Rotating night shift workers (2–7 night shifts per month, usually between 19:00 and 07:00) vs. day workers		x	x				x
[25]	CHE	65	Male	Private and public institutions	Cross-sectional, prospective ^6^	Rotating shift work	Exposure: Mixed shift schedules during at least 6 months with work between 20:00 and 06:00		x	x				x
[26]	GBR	20	Fem.	Healthcare	Cross-sectional, secondary analysis	Rotating shift work	Exposure: Day shifts and ≥2 consecutive night shifts of ≥8 h duration between 20:00 and 08:00							x
[27]	AUS	63	Both	Mixed	Cross-sectional, prospective	Night and rotating shift work	n.s.							x
[28]	BEL	1529	Male	Private and public institutions	Cohort, prospective	Rotating shift work	Exposure groups: Two or three rotating shifts vs. day workers	x	x	x		x	x	
[29]	USA	54,724	Fem.	Healthcare	Cohort, prospective ^7^	Day/evenings only, nights only, early mornings only, rotating with nights, or rotating with no nights	Exposure groups: Day/evenings only, nights only, early mornings only, rotating shifts with nights, rotating shifts with no nights, others/no work - Day shift: Work between 7:00 and 15:00 - Evening shift: Work between 15:00 and 23:00 - Night shift: Work between 23:00 and 07:00 - Early morning: Work between 4:00 and 9:00		x	x	x	x	x	x
[30,31]	USA	177,184; 143,410	Fem.	Healthcare	Cohort, prospective ^7,8^	Rotating shift work	Exposure: At least 3 night shifts per month in addition to day and evening shifts in that month		x	x		x	x	x
[32]	DNK	19,873	Fem.	Healthcare	Cohort, prospective ^9^	Night, evening, or rotating shift work	n.s.		x	x	x	x		x
[33]	CHN	3871	Both	Private companies	Cohort, prospective ^10^	Night shift work: permanent, rotating, or irregular work	Exposure groups: Permanent, rotating, or irregular night shift workers, day shift workers Night shift workers: At least one night between 24:00 and 06:00 per month over 1 year, with subgroups: - Permanent night shift work: Only during nighttime - Rotating shift work: Fixed pattern of daytime and nighttime shifts (e.g., morning, evening, then night shifts, and repeated cycle) - Irregular shift work: No fixed schedule of nights Daytime workers: All not meeting the criteria above		x					x
[34]	NOR	2965	Both	Healthcare	Cohort, prospective ^11^	Day, evening, night, and rotating shift work	Exposure groups: Day shifts only, evening shifts only, two-shift schedule (day + evening), or three-shift schedule (day + evening + night) - Day shift: Work between 07:00 and 15:00 - Evening shift: Work between 14:30 and 22:00 - Night shift: Work between 22:00 and 07:00		x					
[35,36]	NLD	485; 596	Both	Healthcare	Cohort, prospective ^12,13^	Day, evening, night, and rotating shift work	Exposure groups: Day, evening, night, and rotating shift workers Day workers: Only between 08:00 and 17:00, including those who stopped rotating and night shifts ≥ 6 months prior to study in Loef et al. [36] Rotating shift workers: Rotating between day shifts (mostly 07:30–16:00), evening shifts (mostly 15:00–23:00), and night shifts (mostly 23:00–07:30)		x				x	x
[37]	NLD	1061	Both	Healthcare	Cohort, prospective ^14^	Day, evening, night, and rotating shift work	Exposure groups: Day, evening, night or rotating shifts ≥1 year before each wave of the cohort - Evening shift: Work ending before 24:00 - Night shift: Work continuing or starting after 24:00 - Day workers: No past or current shift work		x			x	x	
[38]	Intl	n.a.	Both	Mixed	Systematic review and meta-analysis ^15^	Night shift work	Night shift: Work between 24:00 and 05:00	x	x	x		x	x	
[39]	Intl	n.a.	Both	Mixed	Systematic review and meta-analysis^15^	Rotating shift work and other types of shift work (mixed)	Any work outside of daytime hours	x						
[40]	Intl	n.a.	Both	Mixed	Systematic review and meta-analysis ^15^	n.s.	n.s.	x						
[41]	Intl	n.a.	Both	Mixed	Systematic review and meta-analysis ^15^	Night shift work	Night shift: Work between 24:00 and 05:00		x					
[42]	Intl	n.a.	Both	Mixed	Systematic review and meta-analysis ^15^	n.s.	Any work outside of daytime hours (from 09:00 to 17:00)		x					
[43]	Intl	n.a.	Both	Healthcare	Systematic review and meta-analysis ^15^	n.s.	Any work outside of daytime hours		x					
[44]	Intl	n.a.	Both	Mixed	Systematic review and meta-analysis ^15^	n.s.	n.s.			x				
[45]	Intl	n.a.	Both	Mixed	Systematic review and meta-analysis ^15^	Night, rotating and uncategorized shift work	Night shift: Mixed schedule of day and night work Rotating shift: Non-fixed shift work			x				
[46]	Intl	n.a.	Both	Mixed	Systematic review and meta-analysis ^15^	Rotating shift work and other types of shift work (mixed)	Permanent night shift or rotating shift, or work arrangements differing from daytime hours (from 07:00–08:00 to 17:00–18:00)				x			
[47]	Intl	n.a.	Both	Mixed	Systematic review and meta-analysis ^15^	Night and rotating shift work	n.s.					x		
[48]	Intl	n.a.	Both	Mixed	Systematic review and meta-analysis ^15^	Permanent night shift work or rotating shift work	Rotating shift: 2 shifts of 12 h each, or 3 shifts of 8 h each						x	
[49]	Intl	n.a.	Both	Mixed	Systematic review and meta-analysis ^15^	Night shift work	Night shift: Work including the hour of midnight and ending before 08:00							x
[50]	Intl	n.a.	Both	Mixed	Systematic review and meta-analysis ^15^	Day, evening, night, and rotating shift work	Night shift: Work after 18:00, not lasting >12 h							x
[51]	Intl	n.a.	Both	Mixed	Systematic review	Night and rotating shift work	n.s.							x
[52]	Intl	n.a.	Both	Mixed	Systematic review	Rotating shift work and other types of shift work (mixed)	Any work outside of daytime hours							x

Abbreviations: BMI, Body mass index; BP, Blood Pressure; CVD, Cardiovascular disease; Fem, Female; Gluc, Glucose metabolism; Intl, International (multiple countries); Lip, Lipid profile; Loc, Location (3-letter country code); MetS, Metabolic syndrome; N., Number of participants (for individual studies); n.a., not applicable; n.s., not specified; Nut, Nutrition/eating patterns; Ref, Reference(s). ^1^ See accompanying text in Section 3 and Section 4 for details on the reviewed studies focusing on shift work. ^2^ Study results adjusted for sex, age, BMI, and occupation. ^3^ Study results adjusted for age. ^4^ Study results adjusted for age, BMI, breakfast skipping, physical activity, alcohol consumption, smoking status, marital status, family income, education, sleep, self-reported health status. ^5^ Study results adjusted for age, waist to hip ratio, height, gastro-intestinal diseases, diabetes, concomitant medication, smoking, season, number of children, job duration, chronotype. ^6^ Study results adjusted for total energy intake, the workplace. ^7^ Study results adjusted for age, ethnicity, BMI, metabolic factors (waist circumference, diastolic BP and HDL cholesterol), family history of diabetes, menopausal status, total energy intake, dietary score, physical activity, alcohol consumption, smoking status, concomitant drugs. ^8^ Study results adjusted for age, ethnicity, BMI, family history of diabetes, menopausal status, total energy intake, dietary score, physical activity, alcohol consumption, smoking status, concomitant drugs, marital status, health status. ^9^ Study results adjusted for age, BMI, acute myocardial infarction, hypertension, intake of fatty meat, fruit and vegetables, physical activity, alcohol consumption, smoking status, marital status, employment status. ^10^ Study results adjusted for sex, age, intake of fruit and vegetable, physical activity, alcohol consumption, smoking status, marital status, education, working hours, sleep, mental stress. ^11^ Study results adjusted for sex, age, BMI, marital status, children living at home, education. ^12^ Study results adjusted for sex, age, physical activity, smoking status, marital status, education, occupation, working hours. ^13^ Study results adjusted for sex, age, alcohol consumption, smoking status, education, occupation, self-reported health status. ^14^ Study results adjusted for sex, age, concomitant medication, education, chronotype. ^15^ The meta-analyses listed above included source studies that adjusted for different confounding factors, but also some studies with no adjustment. We refer the reader to the original articles for further details.
